# Antimicrobial Resistance of *Campylobacter coli* Isolated from Caecal Samples of Fattening Pigs at Slaughter

**DOI:** 10.3390/microorganisms11061540

**Published:** 2023-06-09

**Authors:** Triin Tedersoo, Mati Roasto, Mihkel Mäesaar, Maria Fredriksson-Ahomaa, Kadrin Meremäe

**Affiliations:** 1 Chair of Veterinary Biomedicine and Food Hygiene, Institute of Veterinary Medicine and Animal Sciences, Estonian University of Life Sciences, Kreutzwaldi 56/3, 51014 Tartu, Estonia; triin.tedersoo@student.emu.ee (T.T.); mati.roasto@emu.ee (M.R.); mihkel.maesaar@emu.ee (M.M.); 2 National Centre for Laboratory Research and Risk Assessment, Kreutzwaldi 30, 51006 Tartu, Estonia; 3 Food Hygiene and Environmental Health, Faculty of Veterinary Medicine, University of Helsinki, PL 66 (Agnes Sjöbergin katu 2), 00014 Helsinki, Finland; maria.fredriksson-ahomaa@helsinki.fi

**Keywords:** *Campylobacter coli*, antimicrobial resistance, pigs, caecal samples, slaughterhouse

## Abstract

Pigs are known as the main *Campylobacter coli* reservoirs. Campylobacteriosis, the most commonly reported gastrointestinal disease in humans, is mainly caused by the consumption of poultry meat, and little is known about the role of pork. Pigs are often associated with *C*. *coli*, including antimicrobial-resistant isolates. Therefore, the entire pork production chain must be considered as an important source of antimicrobial-resistant *C*. *coli*. This study aimed to determine the antimicrobial resistance of *Campylobacter* spp. isolated from caecal samples of fattening pigs at the Estonian slaughterhouse level over a five-year period. The proportion of *Campylobacter*-positive caecal samples was 52%. All *Campylobacter* isolates were identified as *C*. *coli*. A high proportion of the isolates were resistant to most of the studied antimicrobials. The resistance to streptomycin, tetracycline, ciprofloxacin and nalidixic acid was 74.8%, 54.4%, 34.4% and 31.9%, respectively. In addition, a high proportion (15.1%) of the isolates were multidrug-resistant and, in total, 93.3% were resistant to at least one antimicrobial.

## 1. Introduction

In the European Union (EU), there are approximately 246,000 cases of *Campylobacter* infections per year, mainly due to contaminated food [1]. In general, *Campylobacter* infection is self-limiting, but in the case of immunodeficiency, it can cause very serious health complications, even death [2]. Poultry meat is the main source of human campylobacteriosis, but pork is also considered an important source of *Campylobacter coli*-related human cases [3,4]. The transmission of *Campylobacter* primarily occurs through farm animals and may further contaminate the entire food production chain [5,6]. Pigs are the main reservoir of *C*. *coli* [7,8,9,10] and contaminated pork can pose a health risk for humans. Different studies have found a possible link between human and pig strains [9]. There is also a risk of multidrug-resistant (MDR) *Campylobacter* spreading from animals to humans [11]. MDR is defined as the antimicrobial resistance of microorganisms to three or more unrelated antimicrobials [12]. As pigs are usually subclinically infected with *Campylobacter* spp., there is a high contamination possibility of carcasses during the slaughter process [13]. Intensive farming is associated with the use of antimicrobials, and food-producing animals are often carriers of antimicrobial-resistant (AMR) microorganisms, which can be transmitted to humans via contaminated food [14]. Several studies have raised the importance of AMR *C*. *coli* as an emergent problem in humans [10,15]. Macrolides and fluoroquinolones are the first-choice antimicrobials in campylobacteriosis treatment [16]. According to EFSA and ECDC [17], AMR *Campylobacter* is very common, especially to fluoroquinolones, which are defined as critically important antimicrobials for humans; therefore, their veterinary use should be avoided.

The aim of the study was to determine the occurrence of resistance of *Campylobacter* spp. isolated from caecal samples of fattening pigs at slaughter.

## 2. Materials and Methods

### 2.1. Campylobacter Isolates and Antimicrobial Susceptibility Testing

The sampling was carried out by the veterinary officials of the Agriculture and Food Board (AFB) within the Estonian National Monitoring Programme, according to European Union (EU) Directive 2003/99/EC [18] and the Commission’s implementing decision of 12 November 2013 [19], on the monitoring and reporting of antimicrobial resistance in zoonotic and commensal bacteria. At slaughterhouses, a total of 229 caecal samples representing all Estonian fattening pig farms (one per year) were analysed. Within a five-year period, 87, 68 and 74 samples were collected in 2015, 2017 and 2019, respectively. Direct and enrichment culture methods were used in accordance with instructions laid out in the ISO 10272-1:2018 standard [20]. *Campylobacter* spp. isolation and antimicrobial susceptibility testing were performed at the National Centre for Laboratory Research and Risk Assessment (LABRIS). According to the method, 10 g of pig intestinal contents from the caecum was aseptically taken and placed into a sterile plastic bag, where 90 mL of Bolton broth (Oxoid Ltd.; Hampshire, United Kingdom) was added, and homogenized for one minute in a Stomacher 400 Circulator (Heidolph Instruments GmbH & Co. KG; Schwabach, Germany). The samples were then incubated under microaerobic conditions at 37 °C for 4–6 h, followed by 41.5 ± 0.5 °C for 44 ± 4 h. Further on, 10 µL of the enrichment broth was plated on mCCDA agar (Oxoid Ltd.) and incubated under microaerobic conditions at 41.5 ± 0.5 °C for 44 ± 4 h. Typical *Campylobacter* colonies on mCCDA were streaked on Columbia blood agar (Oxoid Ltd.) plates and then incubated under microaerobic conditions in anaerobic jars with CampGen^TM^ reagents (Oxoid Ltd.) at 41.5 ± 0.5 °C for 24 h. *Campylobacter* was confirmed when colonies were Gram-negative, motile, oxidase-positive and did not grow in aerobic conditions at 41.5 ± 0.5 °C and at 25 °C. *Campylobacter* isolates were stored at −82 °C in cryotubes (Technical Service Consultants Ltd.; Lancashire, UK).

The minimal inhibitory concentration (MIC) values of the isolates were determined against erythromycin, ciprofloxacin, tetracycline, streptomycin, nalidixic acid and gentamicin according to the European Committee on Antimicrobial Susceptibility Testing (EUCAST) recommendations for a EUCAMP2 plate (TREK Diagnostic Systems Ltd.; East Grinstead, UK). Briefly, the *Campylobacter* isolates were first cultured on Columbia blood agar (Oxoid Ltd.) and incubated at 41.5 ± 0.5 °C for 44 ± 4 h at microaerobic conditions. A loopful (1 µL) of bacterial growth was transferred to the test tube containing 2 mL of physiological solution, achieving an estimated inoculum density of 10^8^ CFU/mL. After that, 50 µL of the inoculum was pipetted into the test tube, which contained 10 mL of cation-adjusted Mueller–Hinton broth with 5% of laked horse blood (CAMHB+LHB) (Oxoid Ltd.). Finally, 100 µL of bacterial suspension was transferred into microtiter plates, which were incubated at 35 ± 2 °C under microaerobic conditions in anaerobic jars with CampGen^TM^ reagents (Oxoid Ltd.) for 44 ± 4 h. The MIC was determined as the lowest concentration that completely inhibited campylobacter growth. The purity control of the bacterial suspension was also performed by plating 10 µL of suspension on Columbia agar (Oxoid Ltd.) and incubating it at 37 °C under microaerobic conditions for 40–48 h. Colony counts from 50 to 250 per plate were accepted. The cut-off values recommended by the EUCAST were used for *Campylobacter coli* in accordance with the European Commission implementing decision 2013/652/EU [19] on the monitoring and reporting of antimicrobial resistance in zoonotic and commensal bacteria. The cut-off values for *C*. *coli* were erythromycin >8 µg/mL, ciprofloxacin >0.5 µg/mL, tetracycline >2 µg/mL, streptomycin >4 µg/mL, nalidixic acid >16 µg/mL and gentamicin >2 µg/mL. The quality control was performed according to the EU Reference Laboratory for Antimicrobial Resistance (EURL-AR) instructions and protocols. The strains used for the quality control of *Campylobacter* MIC analyses were *C*. *coli* EURL-AR strain 2012-70-443-2 and *C*. *jejuni* ATCC 33560.

### 2.2. Species Identification

A multiplex PCR assay was used for the identification and differentiation of Campylobacter species according to the standard operating procedure of the LABRIS. Genomic DNA was extracted according to the manufacturer’s instructions from a loopful of bacterial growth from the blood agar using the RTP^®^ Bacterial DNA Mini Kit (STRATEC Molecular GmbH, Berlin, Germany). DNA amplification was conducted with an Eppendorf Mastercycler^®^ ep gradient (Eppendorf AG, Hamburg, Germany), with the following thermal cycling conditions: 95 °C for 6 min, followed by 30 cycles of 95 °C for 30 s, 59 °C for 30 s and 72 °C for 30 s, and a final extension at 72 °C for 7 min. The amplified PCR products were separated in 1.5% agarose gel at 125 V for 55 min, stained in ethidium bromide (Sigma-Aldrich Chemie GmbH, Taufkirchen, Germany) solution and visualized with a ChemiDoc^TM^ XRS+ system (Bio-Rad Laboratories Inc., Hercules, CA, USA).

### 2.3. Statistical Analysis

Microsoft Excel 2010 software (Microsoft Corporation; Redmond, WA, USA) and R v4.2.3 [21] were used to record the results and for statistical analyses, respectively. A logistic regression model, followed by pairwise multiple comparisons with Tukey correction, was used to determine (1) the differences in proportions of *Campylobacter* positive caecal samples between the studied years; (2) the differences in total antimicrobial resistance proportion between 2015, 2017 and 2019; and (3) differences in total antimicrobial resistance between specific antimicrobials in all the years combined. The results were considered statistically significant at *p* < 0.05. Confidence intervals (95% CI) were calculated using a 1-sample proportion test with continuity correction.

## 3. Results

*Campylobacter* spp. positive caecal samples of fattening pigs at the slaughterhouse level in 2015, 2017 and 2019 are shown in Table 1. The proportion of *Campylobacter*-positive caecal samples in 2019 was significantly different (*p* < 0.001) compared to previous years (Table 1). In total, 52% of the caecal samples were *Campylobacter*-positive in the studied period.

The results of antimicrobial susceptibility testing showed that only eight (6.7%) isolates were sensitive to all the tested antimicrobials (Table 2). A total of 111 (93.3%) isolates were resistant to one or more antimicrobials: 55 (46.2%) isolates were resistant to one antimicrobial, 38 (32.0%) were resistant to at least two antimicrobials, and 18 (15.1%) were resistant to three or more antimicrobials not belonging to the same group. The proportion of resistant isolates was extremely high in all the studied years. The resistance to one or more antimicrobials was 87.9%, 95.0% and 95.5% in the years 2015, 2017 and 2019, respectively.

The resistance of *C*. *coli* isolates is presented in Table 3. A very high proportion of isolates were resistant to streptomycin (74.8%) and tetracycline (45.4%), followed by ciprofloxacin (34.4%) and nalidixic acid (31.9%). All isolates found in 2017 and 2019 were sensitive to erythromycin and gentamicin. No statistical differences in the AMR of specific antimicrobials between different years were found; however, the resistance to fluoroquinolones and tetracycline increased slightly in the study period. In 2015, the resistance to ciprofloxacin was 27.3%, followed by 30.0% and 39.4% in 2017 and 2019, respectively. During the same period, tetracycline resistance ranged from 36.4% to 51.5%. Throughout the study period, the resistance to streptomycin remained extremely high with the highest proportion (85.0%) in the year 2017. Statistically significant differences (*p* < 0.05) in the proportion of AMR between specific antimicrobials were found, except for erythromycin and gentamicin, ciprofloxacin and tetracycline/nalidixic acid, and nalidixic acid and tetracycline. Resistance against streptomycin was significantly higher compared to the other five antimicrobials tested in our study (Table 3).

In total, 33.6% of *C*. *coli* isolates were resistant only to streptomycin, 17.6% to a combination of tetracycline/streptomycin and 13.4% to ciprofloxacin/tetracycline/nalidixic acid/streptomycin. The detected resistance phenotypes are presented in Table 4.

The highest MIC values were exceeded for 54.6%, 20.2%, 17.7% and 5.9% of *C*. *coli* isolates against streptomycin, nalidixic acid, tetracycline and ciprofloxacin, respectively. Conversely, 99.2% of the isolates were sensitive to erythromycin and gentamicin (Table 5). In total, 93.3% of *C*. *coli* isolates were resistant to at least one antimicrobial and 15.1% of the isolates were MDR. A total of 16 (13.4% [95% CI: 8.1–21.2]) MDR isolates demonstrated a phenotypic combination of ciprofloxacin/tetracycline/nalidixic acid/streptomycin (Table 4).

## 4. Discussion

*C*. *coli* originating from pigs has been associated with human *Campylobacter* infections [22,23]. Pork is the most consumed meat in Estonia with 42 kg per inhabitant in 2021 [24]; therefore, the presence of AMR *Campylobacter* in pork has a potential public health impact. Among foodborne pathogens, the prevalence and AMR of *Campylobacter* in poultry [25,26] and the prevalence of *Salmonella* in the Estonian pork production chain [27] are well studied, but there are no studies on the prevalence and AMR of *Campylobacter* in fattening pigs in Estonia.

In this study, we have gathered the information on the AMR of *C. coli* isolated from fattening pigs at the Estonian slaughterhouse level within a five-year period. We found that 52% of the caecal samples of fattening pigs collected at the slaughterhouse level were positive for *Campylobacter*. However, compared to the proportions of positive samples, Pezzotti et al. [28] in Italy and Meistere et al. [9] in Latvia found higher *Campylobacter* spp. prevalence in rectal swabs and caecal samples, respectively, 63.5% and 83.3%. In the present study, all isolates detected from caecal samples were identified as *C*. *coli*, which is consistent with other similar studies [8,10,29]. Additionally, slaughterhouse studies in Latvia and Denmark revealed that ~90% of the pig-origin isolates were *C*. *coli* [9,30]. In addition, Wieczorek and Osek [11] in Poland found that *Campylobacter* isolated from pig carcasses were mostly *C*. *coli*.

*C*. *coli* in the intestinal tracts of pigs can easily cause contamination in the entire pork processing chain if there is a lack of effective biosafety and other control measures [5,6]. There are few examples of very low *Campylobacter* prevalence or even of *Campylobacter*-free pig herds available in the literature. In Norway, specific-pathogen-free (SPF) pig herds were studied, and it was found that the intervention of *Campylobacter* at the herd level is possible because some SPF herds tested *Campylobacter* negative both in autumn and summer [31]. Lindblad et al. [32] demonstrated a very low (1.0%) prevalence of *Campylobacter* in pig carcasses in Swedish slaughterhouses.

AMR is a global public concern because resistant pathogens might compromise the treatment of infections and may even result in the death of animals and humans. Unfortunately, the number of *Campylobacter* isolates resistant to critically important antimicrobials (CIAs), especially fluoroquinolones, is increasing [33]. Initially, the monitoring of AMR in *C*. *coli* isolates from caecal samples gathered at slaughter from fattening pigs based on the Member State decision to test *C*. *coli* was laid down by the Commission Implementing Decision 2013/652/EU [19], following Directive 2003/99/EC [18], on the monitoring of zoonoses and zoonotic agents. The antimicrobials included for resistance monitoring of *C*. *coli* isolates were erythromycin, ciprofloxacin, tetracycline, gentamicin, nalidixic acid and streptomycin. In 2020, the Commission Implementing Decision 2020/1729/EU [34] repealed Decision 2013/652/EU [19]. Due to the amended Decision, nalidixic acid and streptomycin were removed, and chloramphenicol, together with ertapenem, was added to the list of antimicrobials included in the AMR monitoring of *C*. *coli* from fattening pigs. As requested by the EU legislation, EUCAST thresholds for resistance need to be followed [19,34]. In this study, the antimicrobials given in Implementing Decision 2013/652/EU [19] were used because it was applicable for the period 2015–2019. In recent years, AMR monitoring for pig-origin *C*. *coli* isolates has not been carried out in Estonia.

The present study found a very high proportion of resistant *C*. *coli* isolates throughout the study period in pig caecal samples at slaughter. According to the Estonian Agency of Medicines [35], the most used antimicrobials in pigs and cattle in 2022 were doxycycline, tiamulin and amoxicillin. Of the overall sale proportion, the most sold antimicrobial classes used for veterinary purposes in Estonia in 2022 were tetracyclines, penicillins and pleuromutilins, respectively, 25.5%, 22.3% and 18.4% [35].

In the present study, a high proportion of isolates was resistant to streptomycin (74.8%), tetracycline (45.4%) and fluoroquinolones (34.4%). Similarly, a Latvian study [9] found an extremely high proportion of streptomycin-resistant *C*. *coli* isolated from pig caecal samples. In addition, in Italy, Pezzotti et al. [28] found that *C*. *coli* isolates of pig origin were highly resistant to tetracycline and streptomycin. A lower resistance against these antimicrobials was observed in Finland [36] and Denmark [37]. Since 2009, the use of CIAs in Danish pig herds has been phased out and the use of tetracyclines in pigs has decreased significantly [38].

Fluoroquinolones (e.g., ciprofloxacin) are categorised as CIA [39] and are used as a first-line treatment for invasive human infections. Therefore, the resistance to fluoroquinolones among *Campylobacter* isolates needs special attention. According to the European Union Summary Report [17], the *C*. *coli* resistance to ciprofloxacin was high to extremely high (>70%) in human isolates and very high to extremely high in isolates from fattening pigs (51.7%) [17]. In research studies, different levels of fluoroquinolone resistance among pig *Campylobacter* isolates, e.g., in Finland 18.3% and 34.0% [36,40], in Latvia 53.5% [9] and in Poland 57.1% [11], have been found. In a recent Italian study [10], *C*. *coli* isolates of pig origin were extremely resistant both to quinolones (74.7%) and fluoroquinolones (70.1%). In our study, the resistance to quinolones (nalidixic acid) and fluoroquinolones (ciprofloxacin) was 31.9% and 34.4%, respectively.

According to the World Health Organization’s (WHO’s) medically important antimicrobials list [39], erythromycin is categorized as CIA. In the present study, the resistance against erythromycin was very low (0.8%) with only one resistant isolate. Similar findings have been reported from our neighbouring countries Finland and Latvia [9,36]. A study conducted in Finland showed that tylosin treatment in pigs caused an increase in *C*. *coli* resistance to erythromycin [41]. However, according to Finnish AMR monitoring, erythromycin resistance in *C*. *coli* isolates from Finnish pigs occurs very rarely [40].

MDR among zoonotic isolates needs special attention because they are known as the most serious global public health threats of this century [42,43]. In the present study, 15.1% of *C*. *coli* isolates were MDR, which was defined as the resistance of the isolate to three or more unrelated antimicrobials [12]. MDR is an alarming trend among *C*. *coli* [16,40,44,45]. In the EU, a total of 9.7% of *C*. *coli* isolates from fattening pigs were reported as being MDR [17]. The high proportion of MDR *C*. *coli* isolates indicates an overuse of antimicrobials in animal husbandry [46]. To decrease the use of antimicrobials in food-producing animals and minimize the emergence and spread of MDR bacteria, the implementation of good farm and veterinary practices and application of strict biosecurity measures are necessary [47,48,49]. Additionally, according to Albernaz-Gonçalves et al. [50], the use of antimicrobials and the risk of AMR can be reduced by providing good nutrition, clean water and appropriate living conditions. The WHO has coordinated a global action plan to limit the use of antimicrobials in food-producing animals and to promote responsible use practices to reduce the development of AMR [51]. The World Organisation for Animal Health (OIE) has developed standards on AMR and the use of antimicrobials that also support the global action plan [52]. At the beginning of the year 2022, two important EU regulations came into force promoting the responsible use of antimicrobials in animals. The Veterinary Medicinal Products Regulation (EU) 2019/6 [53] imposes restrictions on the use of certain antimicrobials and promotes the use of alternatives. This regulation also strengthens the monitoring of antimicrobial use in animals, aiming to prevent the development and spread of AMR. In addition, the Regulation (EU) 2019/4 [54] on the manufacture, placement on the market and use of medicated feed states that “Medicinal treatments, especially with antimicrobials, should never replace good husbandry, bio-security and management practices”. To reduce the use of antimicrobials, there is a need to encourage research and innovation to develop new antimicrobials, vaccines and alternative treatments [55]. According to the reports of the Estonian Health Board, the proportion of *C. coli* isolates among human campylobacteriosis cases in Estonia in 2015–2019 was in the range of 6.0–13.4% [56]. In the mentioned period, the year 2018 had the highest proportion (13.4% [95% CI: 10.3–17.2]) of *C. coli* infections. This was related to three *C. coli* outbreaks with nine cases combined. It is important to note that during 2015–2019, the MDR among *C. coli* was eight times higher than in *C. jejuni* among human clinical isolates in Estonia [56]. Between 2014 and 2021, *Campylobacter* was the most common human intestinal pathogen, with *C. jejuni* accounting for approximately 90% of cases and *C. coli* for less than 10% [57]. According to the European Union Summary Report on Antimicrobial Resistance in zoonotic and indicator bacteria from humans, animals and food in 2020–2021 [17], the average level of ciprofloxacin resistance in the EU was 65.8% and 69.6% for *C. coli* human isolates in the years 2020 and 2021, respectively. The highest levels of resistance (100%) to ciprofloxacin in *C. coli* human isolates were reported for Portugal and Estonia. In addition, the resistance to ciprofloxacin in food-producing animals was estimated to be high to extremely high, ranging from 41.7% to 80.4% in the EU. *C. coli* isolates of fattening pigs exhibited 51.7% resistance to ciprofloxacin in the reported countries in 2021. In addition, an extremely high level (70.3%) of resistance to tetracycline was detected in *C. coli* isolates from humans in 2021. The levels of combined resistance to both ciprofloxacin and erythromycin, which are considered critically important for the treatment of campylobacteriosis, was 25.9% in human *C. coli* isolates in Estonia. This was the second highest level of this particular combined resistance after Portugal at the EU level in 2021 [17]. In summary, due to the high proportion of *C*. *coli*-positive caecal samples and the high resistance of *C*. *coli* isolates to studied antimicrobials, the consumption of contaminated pork should be considered a public health risk in Estonia. There is a need to perform source attribution studies to identify the main sources of *C*. *coli* human infections because no studies have been performed yet on the topic. In addition, investigating risk factors on pig farms and conducting awareness campaigns for consumers on how to avoid *Campylobacter* infection are needed.

## 5. Conclusions

In Estonia, approximately 10% of human campylobacteriosis cases are related to *C. coli* and in the year 2022 there were 15.1 campylobacteriosis cases per 100,000 inhabitants. The current study found that pigs are reservoirs of AMR *C*. *coli*; therefore, the consumption of contaminated pork is a potential public health risk. The thorough monitoring and control of the use of antimicrobials at the farm level are extremely important. Due to the high proportion of resistance, there is a need to limit the use of critically and highly important antimicrobials such as fluoroquinolones and macrolides in the treatment of pigs and to improve the monitoring and control of antimicrobial use in farm animals.

## Figures and Tables

**Table 1:** *Campylobacter coli* in caecal samples of fattening pigs at the slaughterhouse level ^1^.

Year	No. of Samples ^2^	No. of Positive Samples (%)	95% CI ^3^
2015	87	33 (37.9)	27.9–49.0
2017	68	20 (29.4)	19.3–41.9
2019	74	66 (89.2)	79.3–94.9
Total	229	119 (52.0)	45.3–58.6

^1^ National monitoring programme. ^2^ One sample per farm per year. ^3^ 95% confidence interval.

**Table 2:** The number of resistant and susceptible *Campylobacter coli* isolates from caecal samples of fattening pigs at the slaughterhouse level.

Year	No. of Isolates	Antimicrobial-Resistant Isolates (No.)				Multidrug-Resistant ^1^	Susceptible to All Antimicrobials (%)
		Resistant to One	Resistant to Two	Resistant to Three	Resistant to Four		
2015	33	17	7	4	1	5	4 (12.1)
2017	20	10	6	3	-	3	1 (5.0)
2019	66	28	25	10	-	10	3 (4.5)
Total	119	55	38	17	1	18	8 (6.7)

^1^ Number of isolates resistant to three or more unrelated antimicrobials.

**Table 3:** Resistance of *Campylobacter coli* isolates to studied antimicrobials.

Antimicrobial	Number of Resistant Isolates/All Isolates Tested (%)			
	2015	2017	2019	All ^1^
Erythromycin	1/33 (3.0)	0/20 (0)	0/66 (0)	1/119 (0.8) ^a^
Ciprofloxacin	9/33 (27.3)	6/20 (30.0)	26/66 (39.4)	41/119 (34.4) ^b^
Tetracycline	12/33 (36.4)	8/20 (40.0)	34/66 (51.5)	54/119 (45.4) ^b,c^
Gentamicin	1/33 (3.0)	0/20 (0)	0/66 (0)	1/119 (0.8) ^a^
Nalidixic acid	8/33 (24.2)	4/20 (20.0)	26/66 (39.4)	38/119 (31.9) ^b,c^
Streptomycin	24/33 (72.7)	17/20 (85.0)	48/66 (72.7)	89/119 (74.8) ^d^

^1^ Superscripts with the same letter did not differ significantly (*p* > 0.05) from each other.

**Table 4:** Antimicrobial resistance patterns of *Campylobacter coli* isolated from caecal samples of fattening pigs.

Antimicrobial Resistance Phenotype ^1^	Number of *C. coli* Isolates (%)			Number of All Isolates (%) ^2^
	2015	2017	2019	
Ery/Cip/Tet/Nal/Str	1 (3.0)	0 (0)	0 (0)	1 (0.8)
Cip/Tet/Nal/Str	3 (9.1)	3 (15.0)	10 (15.2)	16 (13.4)
Cip/Tet/Nal	2 (6.1)	0 (0)	5 (7.6)	7 (5.9)
Cip/Nal/Str	1 (3.0)	2 (10.0)	6 (9.1)	9 (7.6)
Cip/Tet/Str	1 (3.0)	0 (0)	0 (0)	1 (0.8)
Cip/Nal	1 (3.0)	0 (0)	5 (7.6)	6 (5.0)
Tet/Str	4 (12.1)	3 (15.0)	14 (21.2)	21 (17.6)
Cip/Tet	0 (0)	1 (5.0)	0 (0)	1 (0.8)
Cip/Str	0 (0)	1 (5.0)	0 (0)	1 (0.8)
Tet	1 (3.0)	1 (5.0)	5 (7.6)	7 (5.9)
Gen	1 (3.0)	0 (0)	0 (0)	1 (0.8)
Str	14 (42.4)	8 (40.0)	18 (27.3)	40 (33.6)
Resistant to one or more antimicrobials	29 (87.9)	19 (95.0)	63 (95.5)	111 (93.3)
Susceptible to all antimicrobials	4 (12.1)	1 (5.0)	3 (4.5)	8 (6.7)
Total	33 (100)	20 (100)	66 (100)	119 (100)

^1^ Tested antimicrobials: Ery, Erythromycin; Cip, Ciprofloxacin; Tet, Tetracycline; Nal, Nalidixic acid; Str, Streptomycin; Gen, Gentamicin. ^2^ The number of resistant isolates in total was 111. Phenotypes of antimicrobial-resistant isolates have been calculated based on 119 isolates.

**Table 5:** The minimum inhibitory concentrations (MIC) of *Campylobacter coli* isolates.

No. of Isolates	Antimicrobial ^1^	No. of Isolates with MIC Value (µg/mL)										
		0.12	0.25	0.5	1	2	4	8	16	32	64	128
119	Ery	-	-	-	113	3	2	-	-	-	-	1 ^(1)^
	Cip	58	17	4	1	4	-	13	22 ^(7)^	-	-	-
	Tet	20	-	40	5	-	1	1	1	4	47 ^(21)^	-
	Gen	10	4	19	60	25	-	-	1 ^(1)^	-	-	-
	Nal	-	-	-	3	4	12	44	18	7	31 ^(24)^	
	Str	-	-	-	-	2	28	20	69 ^(65)^			

^(no)^ The number of *C. coli* isolates with MIC values exceeding the EUCAMP2 maximum concentration. ^1^ Tested antimicrobials: Ery, Erythromycin; Cip, Ciprofloxacin; Tet, Tetracycline; Nal, Nalidixic acid; Str, Streptomycin; Gen, Gentamicin. Solid vertical lines indicate breakpoints between sensitive and resistant isolates. The cut-off values: Nal > 16 µg/mL; Cip > 0.5 µg/mL; Tet > 2 µg/mL; Str > 4 µg/mL; Ery > 8 µg/mL; Gen > 2 µg/mL.

## Data Availability

The data presented in this study are available within the article.
